# Racial and ethnic disparities in postnatal growth of infants born before 30 weeks of gestation

**DOI:** 10.21203/rs.3.rs-7347656/v1

**Published:** 2025-09-01

**Authors:** Fu-Sheng Chou, Hung-Wen Yeh, Crystal Hsueh, Susana Plascencia, Neil Rowen, Michael Chang, Reese Clark, Ashwini Lakshmanan

**Affiliations:** Kaiser Permanente Riverside Medical Center; Children’s Mercy Research Institute; University of California, Los Angeles; Kaiser Permanente Bernard J. Tyson School of Medicine; Kaiser Permanente Bernard J. Tyson School of Medicine; Kaiser Permanente Riverside Medical Center; Pediatrix Center for Research, Education, Quality, and Safety; Kaiser Permanente

## Abstract

**Objective:**

To assess racial and ethnic disparities in birth weight (BW), time to BW regain, and mean growth velocity during the accelerated weight gain phase.

**Study Design:**

A retrospective study was conducted using a de-identified dataset from the Pediatrix Medical Group from 2010 to 2020. The dataset categorizes infants into five racial and ethnic groups: Asian, Hispanic, Black, White, and Other, based on maternal report. The study included infants born between 23 and 29 weeks’ gestation and excluded infants without weight data.

**Results:**

This study included 76,145 infants. Antenatal confounders were balanced using inverse propensity weighting. Black infants had lowest, while Hispanic infants had higher BW z-scores, when compared to White infants. Black infants took the shortest time to regain BW. Hispanic infants had the lowest mean growth velocity.

**Conclusion:**

Racial and ethnic disparities in postnatal growth of infants born before 30 weeks of gestation manifest differently across growth phases.

## Introduction

Over recent decades, significant racial and ethnic disparities in birth outcomes- such as low birth weight (BW), preterm births, and infant mortality- have emerged as a pressing public health challenge.^1,2^ The literature highlights that within the preterm population, there are racial and ethnic disparities in neonatal outcomes, including mortality, morbidity, and the use of breastmilk upon discharge from neonatal intensive care units (NICUs).^3–5^ Emerging evidence further highlights disparities in birth size (an estimate of intrauterine growth) and postnatal growth in preterm infants.^6–9^ Nonetheless, these studies face methodological issues, including inadequate adjustment for race/ethnicity confounders, limited sample sizes, and reliance on indirect methods to estimate growth velocity (GV). However, given that race and ethnicity are social constructs that reflect evolving sociopolitical contexts, exploring disparities in care for preterm infants, specifically regarding intrauterine and postnatal growth, offers insight into unobservable confounders such as racism, oppression, and marginalization.^10–12^ Healthcare providers can only start developing a strategy to reduce health disparities once their existence is clearly understood.

A recent report classified the postnatal growth of preterm infants into three clear phases according to the postnatal weight growth trajectory models: initial weight loss, accelerated weight gain, and stable weight gain.^13^ The initial two phases, covering the period from birth to 34 weeks postmenstrual age (PMA), are especially crucial for the care provided in the NICU. From a health disparity perspective, each phase provides insights into the sources of disparities: differences in BW and time to regain BW may reflect prenatal care inequalities and socio-environmental factors influencing pregnancy outcomes, while variations in the accelerated weight gain phase may highlight institutional or personal biases in monitoring growth and nutrition management.^14^ Building on this framework, the present study investigates racial and ethnic disparities in the dynamic growth of preterm infants across different growth phases. Specifically, this study examines racial and ethnic disparities in BW, time to BW regain, and mean GV during the accelerated weight gain phase, while accounting for antenatal confounders and adjusting for postnatal covariates. The ultimate goal of identifying phase-specific growth disparities is to inform the design of targeted intervention strategies that promote growth equity in the NICU.

## Methods

### Study design and data source

We conducted a retrospective cohort study using a de-identified dataset from the Pediatrix Medical Group Clinical Data Warehouse (CDW).^15^ Based on maternally reported race and ethnicity, this dataset categorizes infants into five groups: Asian, Hispanic, non-Hispanic Black, non-Hispanic White, and Other (not reported as any of the other four groups). Although it does not meet the minimum race/ethnicity category standard issued by the Office of Management and Budget (seven categories instead of five in our dataset)^16^, this dataset still offers valuable insights into phase-specific racial and ethnic disparities in postnatal growth. In all analyses, White served as the reference group. The study included infants born between 23 and 29 weeks of gestation, excluding those without weight data. Additionally, infants with race/ethnicity-specific birth weight z-scores greater than 3 or less than − 3 were excluded.

To balance antenatal confounders, we generated a synthetic weighted population using inverse propensity score weighting. Propensity scores were calculated through generalized boosted regression modeling to estimate the population average treatment effect.^17^ Variables included in the propensity score estimation were potential demographic and antenatal confounders including maternal age, GA, infant sex, maternal obesity, maternal hypertensive disorders of pregnancy (preeclampsia, eclampsia, or HELLP syndrome), fetal growth restriction as determined by obstetricians, maternal smoking during pregnancy, placental abruption, maternal diabetes mellitus, chorioamnionitis, and major congenital anomalies in the fetus.

#### Intrauterine weight growth reference chart development

We developed race- and ethnicity-specific intrauterine weight growth charts using BW data from infants born between 22 0/7 and 41 6/7 weeks of gestation admitted to US Pediatrix NICUs from 2010 to 2022. Separate charts were created for males and females. Generalized additive models for location, scale, and shape (GAMLSS) were applied to model the data against GA in days, using penalized B-splines as the curve smoother. The L (lambda, representing skewness), M (mu, representing the mean), and S (sigma, representing the variance) values for each gestational day were extracted from the model to construct the LMS table. The *gamlss* package in R was used for model development.^18^

#### Postnatal weight growth trajectory modeling

Following the previously described modeling technique—generalized additive mixed models, we modeled postnatal weight growth trajectories for each racial and ethnic group using repeated weight measurements from birth until NICU discharge or 44 weeks PMA if the infants remained admitted.^13^ Infants with the same rounded gestational week and sex were grouped together for model development.

#### Assessing disparities in BW

This analysis included all infants who met the selection criteria. BW z-scores for each infant were calculated using birth-size reference charts specifically designed for White infants. A mixed-effects regression model was created employing the weighted population to assess the correlation between race/ethnicity and BW z-scores. To accommodate variations at the facility level, the facility variable was included as a random effect.

#### Assessing disparities in the time it takes to regain BW

This analysis included only a subset of infants who had daily weight data from day of life (DOL) 0 to 27 and regained their birth weight (BW) by DOL 27. A mixed-effects regression model was created utilizing the weighted population to investigate the relationship between race/ethnicity and the duration needed to regain BW, while accounting for facility-level differences by incorporating the facility variable as a random effect. Birth weight z-scores computed using birth-size reference charts for White infants (considered a confounder) or race/ethnicity-specific charts (considered a covariate), and 1- and 5-minute APGAR scores were included as fixed-effect terms for adjustment.

### Assessing disparities in mean growth velocity between DOL10 and 34 weeks PMA

From the subset used to assess time-to-BW-regain, we further excluded infants whose weight nadir occurred after DOL10 for this analysis. Mean GV between DOL10 and 34 weeks PMA was calculated for each infant using the accurate standard method as mentioned in Patel et al.^19^ (also known as the “Daily” method described by Fenton et al.^20^), employing the following formula:

$$\text{mean GV}=\frac{1}{\left(n-1\right)}\sum_{i=0}^{n-1}\frac{\left[\text{Weight on DOL}\;(i+1)-\text{Weight on DOL}(i)\right]}{\left[\frac{\left(\text{Weight on DOL}(i)+\text{Weight on DOL}\;(i+1\right)}{2}\right]}(g/kg/day),$$

where *n* represents the total number of days, with the date of birth designated as DOL0. A mixed-effects regression model was developed using the weighted population to evaluate the association between race/ethnicity and mean GV, accounting for facility-level variations by including the facility variable as a random effect. Neonatal morbidities, including grade 3/4 intraventricular hemorrhage (IVH), patent ductus arteriosus (PDA) requiring treatment, grade 2/3 bronchopulmonary dysplasia (BPD), stage 2–5 retinopathy of prematurity (ROP), receiving treatment for ROP, medically treated or surgically treated necrotizing enterocolitis (NEC), intestinal perforation, postnatal corticosteroids use for BPD, and total antibiotic days after DOL 3 were included as fixed-effect terms for adjustment.

### Statistical Analysis

Continuous variables were presented as either the mean with standard deviation or the median with interquartile range for both the unweighted cohort and the weighted population. Statistical comparisons were performed using parametric or non-parametric analysis of variance tests, as appropriate. Categorical variables were reported as numbers (percentages), with statistical comparisons conducted using the chi-squared test. For the weighted population, all weighted estimates were calculated using weighted estimation functions from the *survey* package v4.4–2 for R.^21^

Raw data of the outcome variables were transformed to follow a normal distribution using the ordered quantile technique prior to model development using the *bestNormalize* package v1.9.1 for R.^22^ Once the models were constructed, estimates and 95% confidence intervals (CIs) for each racial/ethnic group were extracted and inversely transformed to the original scale. The covariate parameters used for these calculations are detailed in Supplemental Table 1.

## Results

From the Pediatrix CDW, we identified a cohort of 76,550 infants born between 2010 and 2020 who met the inclusion criteria. Of these, 34 infants did not have weight data, and 371 outliers, with a BW z-score > 3 or < −3, were identified. The final cohort consisted of 76,145 infants (Supplemental Fig. 1). Confounders were balanced using inverse propensity weighting (Table 1). The respective numbers of infants included in each analysis and the corresponding weighted populations are shown in Supplemental Fig. 1.

### Assessing disparities in BW

**Figure 1:**
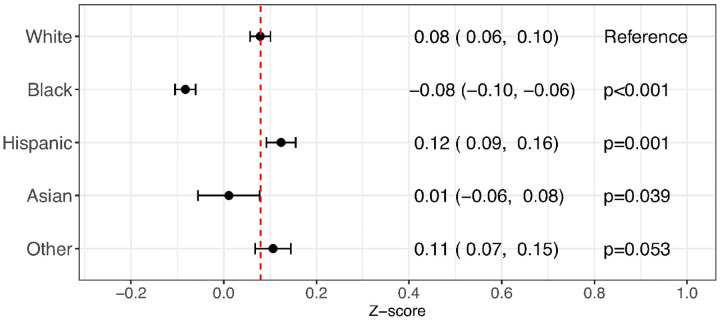
Regression analysis estimating racial and ethnic disparities in birth weight z-scores. A mixed-effects model was developed to assess racial and ethnic disparities in birth weight z-scores using all infants included in this study. The birth weight z-scores were calculated using birth-size reference charts for White infants created as part of this study.

The BW z-score estimate of White infants from the weighted population was 0.08 (95% CI: 0.06 to 0.10). Using White infants as the reference, Black and Asian infants had significantly lower BW, with estimated mean z-scores of −0.08 (−0.10 to −0.06) and 0.01 (−0.06 to 0.08), respectively. In contrast, Hispanic infants had significantly higher BW, with an estimated mean z-score of 0.12 (0.09 to 0.16) (Fig. 1).

### Assessing disparities in the time to BW regain

In this analysis, we further excluded 35,317 infants who either lacked daily weight data or had regained BW after day of life 27. A comparison of the antenatal and postnatal characteristics between the included and excluded infants from the weighted population revealed that both groups are comparable (a standardized mean difference [SMD] < 0.1) except that the excluded group had a higher rate of grade 3/4 IVH, with an SMD of 0.262 (Supplemental Table 2).

**Figure 2:**
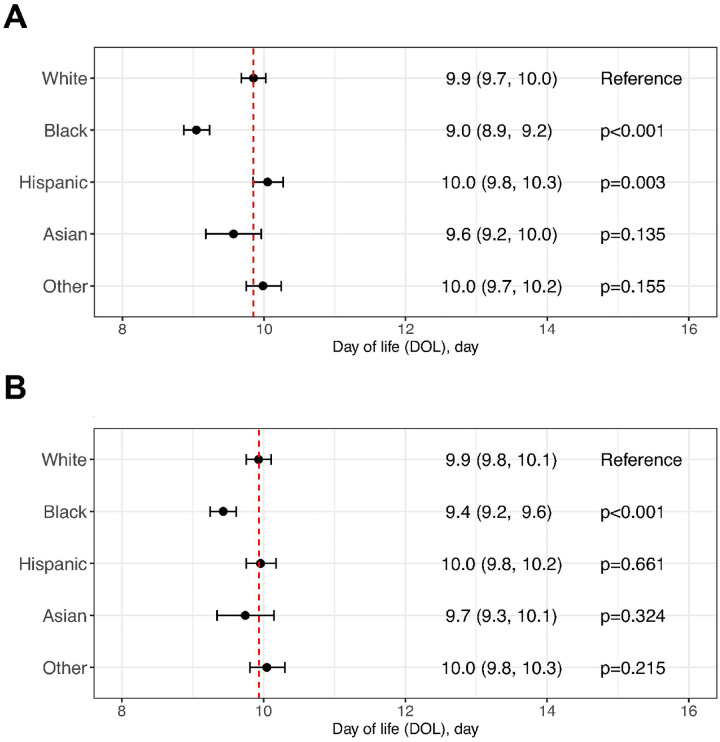
Regression analysis estimating racial and ethnic disparities in time to birth weight regain. A mixed-effects model was developed to assess racial and ethnic disparities in time to birth weight regain, based on a weighted subpopulation of infants who had daily weight measurement recorded and regained their birth weight by day of life 27. The model was adjusted for APGAR scores at 1 and 5 minutes and birth weight z-scores, calculated using (A) race/ethnicity-specific birth-size reference charts or (B) birth-size reference charts for White infants created for this study. Time to birth weight regain for White infants was used as the reference.

We first performed the analysis by including BW z-score as a covariate (using racial/ethnic-specific birth-size reference charts for BW z-score calculation). We found that it took White infants an estimated 9.9 (9.7 to 10.0) days to regain BW. In comparison, Black infants took significantly less time to regain BW, with an estimated 9.0 (8.9 to 9.2) days, while Hispanic infants took significantly longer to regain BW, with an estimated 10.1 (9.8 to 10.3) days. Asian and Other infants (those infants not assigned to the previous racial/ethnic categories) took a comparable amount of time to regain BW—9.6 (9.2 to 10.0) days for Asian infants and 10.0 (9.7 to 10.2) days for Other infants. In a *post-hoc* analysis, we assessed whether BW z-scores confounded the time it took to regain BW (Fig. 2B). We calculated BW z-scores using the birth-size reference charts for White infants (to treat BW z-scores as a confounder). We found that Black infants continued to take significantly less time to regain BW compared to White infants, with an estimated 9.4 (9.2 to 9.6) days for Black infants compared to 9.9 (9.8 to 10.1) days for White infants. In contrast, when adjusted for size at birth using birth-size reference charts for White for z-score calculation, Hispanic infants no longer took significantly more time to regain BW, with an estimated 10.0 (9.8 to 10.2) days. Adjusting for birth size did not change the results for Asian and Other infants.

### Assessing disparities in mean GV

**Figure 3:**
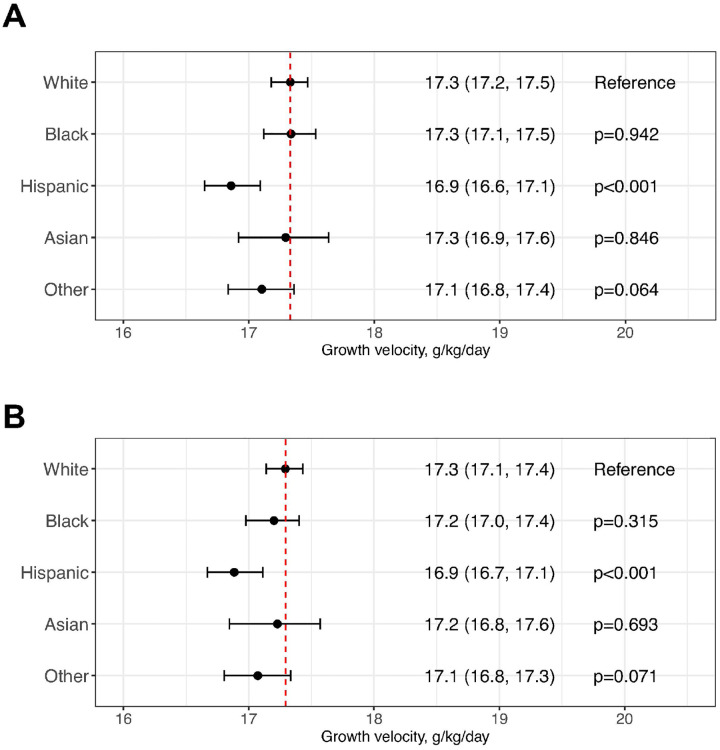
Regression analysis estimating racial and ethnic disparities in mean growth velocity before day of life (DOL) 10 and 34 weeks postmenstrual age. A mixed-effects model was developed to assess racial and ethnic disparities in mean growth velocity using a weighted subpopulation of infants with a weight nadir that occurred by DOL10 and birth weight regained by DOL27 (Figure 1). Mean growth velocity was calculated based on daily weight measurements from DOL 10 to the last recorded weight between 33–34 weeks postmenstrual age. The model was adjusted for several covariates: APGAR scores at 1 and 5 minutes, birth weight z-scores calculated using (A) race/ethnicity-specific birth-size reference charts or (B) birth-size reference charts for White infants created for this study, bronchopulmonary dysplasia (grade 2/3), retinopathy of prematurity (stage 2–5), retinopathy of prematurity requiring treatment, patent ductus arteriosus requiring treatment, medical or surgical necrotizing enterocolitis, intestinal perforation, grade 3/4 intraventricular hemorrhage, total days on antibiotics after DOL 3, and postnatal corticosteroid use. Mean growth velocity for White infants was used as the reference.

By incorporating BW z-scores as a covariate, computed using race/ethnicity-specific charts, we estimated the mean GV for White infants to be 17.3 (17.2 to 17.5) g/kg/day from DOL10 to 34 weeks PMA (Fig. 3A). In contrast, the mean GV for Hispanic infants was significantly lower, at 16.9 (16.6 to 17.1) g/kg/day. Significant differences were not found in other racial and ethnic groups. In a *post-hoc* analysis, we investigated whether the BW z-scores from charts developed for White infants influenced the GV mean differences. This analysis showed that White infants had an adjusted mean GV of 17.3 (17.1 to 17.4) g/kg/day (Fig. 3B). Compared to White infants, Hispanic infants’ mean GV remained significantly lower at 16.9 (16.7 to 17.1) g/kg/day. No significant differences were noted in other racial/ethnic groups.

#### Comparing weight trajectories among racial/ethnic groups

**Figure 4:**
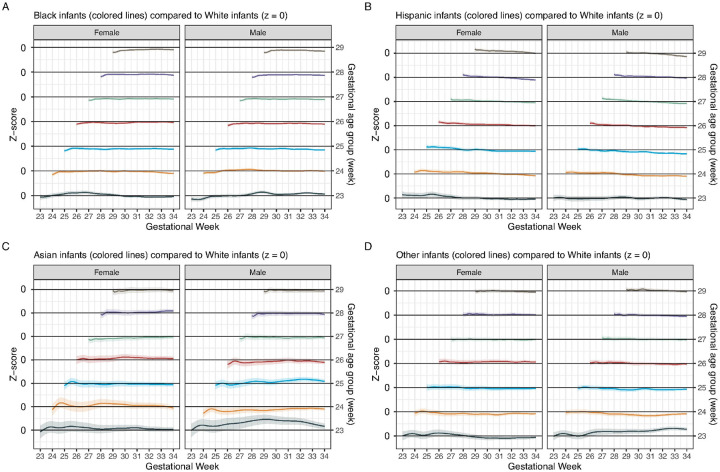
Generalized additive mixed models of postnatal weight growth for each racial/ethnic and sex group. The models were developed separately for each sex and rounded gestational week group. The curves shown here are weight z-score trajectory curves for (A) Black, (B) Hispanic, (C) Asian, and (D) Other (those that could not be categorized into any of the previous categories) infants. Modeled weight trajectory estimates for Black, Hispanic, Asian, and Other infants were converted to z-scores using the postnatal weight growth model for White infants as the reference for calculation. Therefore, the reference line along the z-score of 0 represents White infants. The shades represent standard errors of the trajectory z-scores.

Separate race/ethnicity-specific weight trajectory models were developed using the unweighted cohort. The model estimates were extracted from the respective trajectory models, followed by converting estimates from Black, Hispanic, Asian, and Other infants to z-scores based on the weight trajectory model for White infants. The z-score trajectories were then plotted against PMA for comparison (Fig. 4). Consistent with the phase-specific analyses above, the estimated BW z-scores for Black infants were all < 0. In the first week of life, there was an upward slope in the estimated weight z-score trajectories across GA groups in both sexes, consistent with faster regain of BW. During the subsequent accelerated weight gain phase, the trajectories for Black infants and White infants appeared to be approximately parallel (Fig. 4A).

In contrast, the estimated BW z-scores for Hispanic infants were all > 0. However, the estimated weight z-score trajectories followed a negative slope in all GA groups in both sexes, suggesting slower GV (Fig. 4B). Asian and Other infants appeared to grow similarly to White infants, except in low-GA groups, where the trajectory estimates showed variabilities over time along with wide CIs (Fig. 4C, 4D).

## Discussion

In this study, we investigated racial and ethnic disparities in the dynamic postnatal growth of preterm infants born at less than 30 weeks of gestation. We assessed disparities in: 1) birth weight (BW), which may reflect intrauterine growth; 2) time to BW regain, which may indicate the acuity of illness and the attentiveness to early nutrition; and 3) growth velocity (GV) during the accelerated weight gain phase, which may indicate attentiveness growth surveillance and timely nutrition adjustment. Our goal was to elucidate disparities across different growth phases to identify opportunities for equity in the postnatal growth of very preterm infants. We found that Black infants were smaller than White infants at birth but regained BW more quickly. In contrast, Hispanic infants were born larger than White infants but required a comparable amount of time to regain BW after adjusting for BW z-scores referenced to White. However, Hispanic infants had a significantly lower mean GV during the accelerated weight gain phase. Like Black infants, Asian infants were also born smaller than White infants; however, their subsequent growth trajectory was comparable to that of White infants.

Published studies on racial and ethnic disparities in growth among preterm infants have utilized the 2013 Fenton charts for BW z-score calculation.^7–9^ Although widely utilized, a significant drawback of the Fenton charts lies in their development methodology: a meta-analysis of six studies that used distinct selection criteria and included diverse racial and ethnic groups. Additionally, these studies compared BW and BW z-scores among racial/ethnic groups descriptively, without accounting for antenatal confounders. To properly compare disparities in intrauterine growth, we created race/ethnicity-specific birth-size reference charts using data from a recent cohort of newborns admitted to the NICUs and utilized these charts for referencing and z-score calculation. This approach minimizes bias in referencing across gestational ages (GA) due to varying racial/ethnic group compositions. Moreover, the race/ethnicity-specific growth charts for this study were developed by modeling BW against gestational day, enhancing the accuracy of z-score assignments. The LMS tables for the birth-size reference charts specific to each racial and ethnic group are provided in Supplemental Tables 3–12.

For postnatal growth comparisons, both studies by Jerome et al.^7^ and Salas et al.^9^ used the exponential method^19^ to estimate GV from two time points that spanned nearly the entire NICU hospitalization. While the exponential model was comparable to the accurate standard^19^ (also known as the Daily method^20^) when calculating average daily GV, it does not offer a detailed understanding of phase-specific growth. Instead of estimating GV, the study by Lee et al. assessed postnatal growth failure (PGF) as a binary outcome, using a predefined cutoff based on the difference between birth and discharge z-scores.^8^ However, the clinical significance of this metric remains unclear, as the literature on the correlation between changes in z-scores and childhood outcomes has been inconsistent.^23–38^ In our analysis, we calculated mean GV by averaging daily GV between DOL10 and 34 weeks PMA, a period that corresponds to the accelerated weight gain phase as identified in our recent data-driven modeling of postnatal weight growth using real-world weight measurements from NICUs across the US.^13,39^ As the gold standard method for postnatal growth assessment^19^ and with a strong correlation with neurodevelopmental outcomes^40^, directly comparing GV across racial and ethnic groups during the accelerated weight gain phase provides the most robust evidence of racial and ethnic disparities in postnatal growth.

This is by far the largest study to examine racial and ethnic disparities in intrauterine and postnatal growth among infants born at less than 30 weeks of gestation. The detailed recording of weight measurements, as well as maternal, fetal, and neonatal demographic and morbidity profiles, allowed us to adopt a detailed analytic approach. Nonetheless, the study has limitations. First, although we included the caring facility as a random effect, assuming a unified nutrition practice within each facility, the study did not directly account for differences in nutrition administration among individual infants as detailed nutrition data are not available. Second, while we accounted for variation across facilities, we were unable to address potential geographic differences in the composition of racial and ethnic groups, which might influence growth outcomes on a larger scale. Additionally, the dataset did not include key maternal socioeconomic variables, such as insurance type, maternal education, and neighborhood income level, which could have been used for further adjustment to better understand the mechanisms underlying these disparities. There is also no information on structural racism or marginalization. Furthermore, the clinical significance of the identified disparities is unclear due to the lack of long-term outcome data for correlation. Most importantly, the method used to assign racial and ethnic groups could not be verified. Lastly, as with any administrative dataset, the Pediatrix dataset is susceptible to underreporting and inaccurate reporting, despite internal consistency checks, low rates of missing data, and prospective data entry.

In conclusion, we identified racial and ethnic disparities in intrauterine and postnatal growth among infants born at less than 30 weeks of gestation. Although the differences were small, these findings indicate additional opportunities to promote growth equity in the NICU, particularly in addressing the postnatal growth of Hispanic infants during the accelerated weight gain phase. Neonatal providers might consider utilizing postnatal growth trajectory charts, such as the Postnatal Growth Charts for Preterm Infants (https://nicugrowth.app), for real-time growth monitoring. These tools can help guide targeted nutrition strategies to address and mitigate growth-related disparities.

## Supplementary Material

Supplementary Files

This is a list of supplementary files associated with this preprint. Click to download.

- SupplementalTable3LMSTableforWhiteMales.docx
- SupplementalTable4LMSTableforWhiteFemales.docx
- SupplementalTable7LMSTableforHispanicMales.docx
- SupplementalTable5LMSTableforBlackMales.docx
- SupplementalTable11LMSTableforOtherMales.docx
- SupplementalFigure1.pdf
- SupplementalTable1.docx
- SupplementalTable2.docx
- SupplementalTable8LMSTableforHispanicFemales.docx
- SupplementalTable6LMSTableforBlackFemales.docx
- SupplementalTable10LMSTableforAsianFemales.docx
- SupplementalTable9LMSTableforAsianMales.docx
- SupplementalTable12LMSTableforOtherFemales.docx

## Figures and Tables

**Table 1:** Maternal and neonatal characteristics by racial and ethnic group in the unweighted (top) and the weighted population (bottom).

Group	Total	Non-Hispanic White	Non-Hispanic Black	Hispanic	Non-Hispnaic Asian	Other	p-value
UNWEIGHTED							
**Number**	76145	30749	22417	14487	2263	6229	
**Maternal**							
**Age, median [IQR]**	28 [23, 33]	29 [24, 33]	27 [23, 32]	27 [22, 33]	33 [29, 36]	29 [24, 34]	<0.001
**Smoking, n (%)**	5107 (6.7)	3299 (10.7)	1209 (5.4)	238 (1.6)	23 (1.0)	338 (5.4)	<0.001
**Diabetes mellitus, n (%)**	6731 (8.8)	2543 (8.3)	1782 (7.9)	1393 (9.6)	354 (15.6)	659 (10.6)	<0.001
**Obesity, n (%)**	4822 (6.3)	1906 (6.2)	1792 (8.0)	699 (4.8)	54 (2.4)	371 (6.0)	<0.001
**Hypertensive disorder of pregnancy, n (%)**	11649 (15.3)	5019 (16.3)	3602 (16.1)	1844 (12.7)	271 (12.0)	913 (14.7)	<0.001
**Intrauterine growth restriction, n (%)**	6334 (8.3)	2752 (8.9)	2065 (9.2)	780 (5.4)	220 (9.7)	517 (8.3)	<0.001
**Abruption, n (%)**	7646 (10.0)	3336 (10.8)	2070 (9.2)	1396 (9.6)	195 (8.6)	649 (10.4)	<0.001
**Chorioamnionitis, n (%)**	4308 (5.7)	1653 (5.4)	1352 (6.0)	855 (5.9)	115 (5.1)	333 (5.3)	0.006
**Neonatal**							
**Gestational age, median [IQR]**	27 3/7 [25 4/7, 28 5/7]	27 4/7 [25 6/7, 28 6/7]	27 1/7 [25 2/7, 28 4/7]	27 1/7 [25 3/7, 28 5/7]	27 3/7 [25 5/7, 28 6/7]	27 4/7 [25 5/7, 28 6/7]	<0.001
**Male, n (%)**	40448 (53.1)	16550 (53.8)	11388 (50.8)	7883 (54.4)	1257 (55.5)	3370 (54.1)	<0.001
**Congenital anomaly, n (%)**	10195 (13.4)	3998 (13.0)	2890 (12.9)	2137 (14.8)	315 (13.9)	855 (13.7)	<0.001
**Birth weight z-score, mean (SD)**	0.0 (10)	0.0 (1.0)	−0.2 (0.9)	0.1 (1.0)	0.0 (0.9)	0.1 (10)	<0.001
WEIGHTED							
**Number**	374832	75902	75663	75385	72721	75161	
**Maternal**							
**Age, median [IQR]**	28.0 [23.0, 33.0]	28.0 [23.0, 33.0]	28.0 [23.0, 33.0]	28.0 [23.0, 33.0]	28.0 [24.0, 33.0]	28.0 [23.0, 33.0]	0.842
**Smoking, n (%)**	23911 (6.4)	5096 (6.7)	5014 (6.6)	4780 (6.3)	4147 (5.7)	4874 (6.5)	0.582
**Diabetes mellitus, n (%)**	33054 (8.8)	6688 (8.8)	6670 (8.8)	6629 (8.8)	6420 (8.8)	6646 (8.8)	0.998
**Obesity, n (%)**	23432 (6.3)	4796 (6.3)	4787 (6.3)	4743 (6.3)	4435 (6.1)	4672 (6.2)	0.916
**Hypertensive disorder of pregnancy, n (%)**	56763 (15.1)	11628 (15.3)	11593 (15.3)	11509 (15.3)	10748 (14.8)	11286 (15.0)	0.809
**Intrauterine growth restriction, n (%)**	30475 (8.1)	6321 (8.3)	6292 (8.3)	6095 (8.1)	5618 (7.7)	6148 (8.2)	0.689
**Abruption, n (%)**	37138 (9.9)	7620 (10.0)	7550 (10.0)	7641 (10.1)	6856 (9.4)	7472 (9.9)	0.687
**Chorioamnionitis, n (%)**	21025 (5.6)	4267 (5.6)	4281 (5.7)	4329 (5.7)	4044 (5.6)	4105 (5.5)	0.891
**Neonatal**							
**Gestational age, median [IQR]**	27 3/7 [25 4/7, 28 5/7]	27 3/7 [25 4/7, 28 5/7]	27 3/7 [25 4/7, 28 5/7]	27 3/7 [25 4/7, 28 5/7]	27 3/7 [25 4/7, 28 5/7]	27 3/7 [25 4/7, 28 5/7]	0.799
**Male, n (%)**	199117 (53.1)	40337 (53.1)	40123 (53.0)	40016 (53.1)	38636 (53.1)	40004 (53.2)	0.985
**Congenital anomaly, n (%)**	49958 (13.3)	10154 (13.4)	10070 (13.3)	10139 (13.4)	9495 (13.1)	10100 (13.4)	0.886
**Birth weight z-score, mean (SD)**	0.0 (10)	0.0 (1.0)	−0.1 (0.9)	0.1 (1.0)	−0.1 (0.9)	0.1 (10)	<0.001

## Data Availability

Data pertaining to the Pediatrix Medical Group should be requested through the online portal: https://www.pediatrix.com/for-clinicians/creqs
